# Writing Abilities in Compulsive Prisoners

**DOI:** 10.3389/fpsyg.2021.701941

**Published:** 2021-08-02

**Authors:** Lucas Muñoz-López, Francisca López-Torrecillas, Ignacio Martín, María Blasa Sánchez-Barrera, María del Carmen López-Torrecillas, Francisca Serrano

**Affiliations:** ^1^Consejería de Igualdad, Política Sociales y Conciliación de la Junta de Andalucía, University of Granada, Granada, Spain; ^2^Centro de Investigación Mente, Cerebro y Comportamiento, University of Granada, Granada, Spain; ^3^Departamento de Metodología de las Ciencias del Comportamiento, University of Granada, Granada, Spain

**Keywords:** impulsive, compulsive, prison, PROESC, writing

## Abstract

Research has found links between academic failure and criminal offending and suggest that many incarcerated young people have experienced significant behavioral and learning problems in school, which could result in criminal outcomes and poor academic performance. The objective of this study was to analyse writing disorders in impulsive and compulsive prisoners. The sample was composed of 194 male prisoners, of which 81 had been diagnosed with Antisocial Personality Disorder and 113 with Obsessive Compulsive Personality Disorder. Male participants were recruited at the Granada Prison Center. They completed the Demographic, Crime, and Institutional Behavior Interview; the International Personality Disorder Examination (IPDE); The Symptom Checklist (SCL-90-R) and Assessment Battery of Writing Processes (PROESC in its Spanish acronym). We found that prisoners with writing disorders generally have difficulties in the skills necessary to write properly due to impulsive and compulsive behavior.

## Introduction

Classic studies in the literature have found links between academic failure and criminal offending (Samuelsson et al., 2000, 2003). From this point, more recently studies (Jonesa et al., 2013) have shown that 60% of incarcerated youths have problems with literacy skills or writing abilities. Recently, Kippin et al. (2018) confirmed the high prevalence of previously unidentified language disorders among justice-involved youths. In a similar vein, Green et al. (2018) found that education status affects the mechanics of writing of incarcerated males. Some of the proposed reasons for this link include the low-average to a below-average range of intelligence, low academic performance between the fifth- and ninth-grade levels, and a history of high rates of academic failure and grade retention (Exner, 2020). Accordingly, Gabay (2020) suggests that many incarcerated young people experience significant behavioral and learning problems in school, that is to say, micro-aggressions in primary education settings, and such micro-aggression victimization can result in poor academic and criminal outcomes in young people.

Moreover, those with writing difficulties have deficits in regulating mental operations in writing, which, in turn, leads to longer writing latencies, inter-letter intervals, and writing durations along with a greater number of orthographic errors, signs that are typical of dyslexia (Afonso et al., 2015; Nigro et al., 2015; Suzuki and DeKeyser, 2017; Nicolson and Fawcett, 2019). Dyslexia, however, is caused by multiple genetic and environmental risk factors as well as the interplay between them. At the brain level, dyslexia has been associated with impaired structure and function, particularly in the reading and language networks of the left hemisphere (Benítez-Burraco and Murphy, 2019). The neurocognitive influences on dyslexia are also multifactorial and involve phonological processing deficits as well as weaknesses in other oral language skills and processing speed. Peterson and Pennington (2015) have also examined contextual issues such as how dyslexia manifests across languages and social classes. Such problems with regard to orthographic representations (reading latencies, writing the words, choice between homophones of the same stimulus) have been described by Martínez-García et al. (2019). A number of studies suggest that superior cognitive abilities such as language are linked with compulsive behavior (Brainerd et al., 2020; Piette et al., 2020; Ramey et al., 2020).

Disorders involving emotional dysregulation such as impulsivity and compulsivity are often examined to understand individual differences in personality disorder. In accordance with Morse (2017), a focus on emotion dysregulation, impulsivity and compulsivity is central to understanding how trait impulsivity and compulsivity could contribute toward explaining criminal behaviors. Impulsivity and compulsivity characterize a wide range of disorders, and, in some cases, they appear to overlap; for instance, many disorders can be characterized by both impulsivity and compulsivity, either simultaneously or at different times (Grant and Chamberlain, 2019). Impulsive behaviors can often be controlled, while compulsive behaviors may require more specialized and multifactorial (biological, psychological, and social) interventions, as they are often part of a more serious problem. Impulsivity and compulsivity are traits that are thought to underlie violent behavior (Fitzpatrick et al., 2020; Olver et al., 2020). According to The American Psychiatric Association [American Psychiatric Association (APA), 2013] impulsivity can be defined as the execution of unplanned, rapid actions taken without consideration of the possible negative consequences, whilst compulsivity is defined as the occurrence of repeated behaviors, the goal of which is to reduce or avoid anxiety or distress [American Psychiatric Association (APA), 2013]. Namely, the behavior of impulsive patients aims to alleviate anxiety or discomfort, and to satisfy the desire for pleasure, excitement, or gratification. The behavior of compulsive has an exaggerated sense of threat from the outside world and perform rituals/routines in order to neutralize the threat or reduce harm (Figee et al., 2016; Hollander et al., 2016). Further, compulsive misbehavior is regarded as all behavior that is presented as something planned and/or conscious, and in no case is a spontaneous act (Chamberlain et al., 2018).

The behaviors shown by impulsive patients aim to alleviate anxiety or discomfort, and to satisfy the desire for pleasure, excitement, or gratification. Patients at the end of the compulsive spectrum have an exaggerated sense of threat from the outside world and perform rituals/routines such as obsessive-compulsive behaviors in order to neutralize the threat or reduce harm. This latter point marks compulsive or risk-averse behaviors that are characterized by an overestimation of the probability of future harm. Thus, some compulsive patients engage in behaviors or rituals to achieve short-term benefits (stress relief) in spite of the long-term negative consequences (Figee et al., 2016; Hollander et al., 2016).

Recent advances in the understanding of the neural circuits involved in impulsivity and compulsivity have shown that many psychopathological disorders share these two dimensions (impulsivity and compulsivity). Despite the fact that impulsivity and compulsivity are noted for their role in different aspects of response control, there is a high probability that both are mediated by related neural circuits, albeit linked in different ways to motivational and decision-making processes. For example, according to Suhas and Rao (2019), an increase in frontal lobe activity is associated with compulsive disorders such as obsessive-compulsive disorder (OCD), whilst a decrease in frontal lobe activity is involved in impulsive disorders such as antisocial disorder.

Dyslexia may appear due to damage or malfunction in these neural circuits and also in the areas with which they are connected. The complex functions that are linked to these areas could help to explain the possible relationship between compulsivity and oral and written language pathologies. However, to the best of our knowledge, no studies have yet been conducted to demonstrate the relationship between writing disorders and compulsive and criminal behaviors. Thus, the objective of this study is to analyse writing disorders in impulsive and compulsive prisoners.

## Methodology

### Participants

The current study included 194 men, 81 of which had been diagnosed with ASPD with a mean age of 36.86 years (*SD* = 9.32) and 113 diagnosed with OCPD with a mean age of 38.78 years (*SD* = 8.47). Male participants were recruited at the Granada Prison Center. Participants were screened using the International Personality Disorder Examination (IPDE; Loranger et al., 1994). The inclusion criteria were to be between 18 and 55 years old, suffering from either ASPD or OCPD and to be literate. Participants were excluded according to the following criteria: being older than 55 years, having a physical impairment, psychiatric illness (schizophrenia or depression), neurological disorder, or currently undergoing psychopharmacological treatment (see Tables 1, 2).

**Table 1:** Sociodemographic variables and those related to drug abuse, alcoholism history, alcohol, and drug quit treatment and crimes between groups.

	**ASPD**	**OCPD**	**χ^2^**
**Marital Status (** ***N*** **)**			10,916^**^
Single	41	48	
Married	10	35	
Divorced	12	15	
Widower	1	0	
Convive with couple	17	15	
**Level educational (** ***N*** **)**			1,575
Not elementary	17	16	
Elementary	33	51	
Secondary	21	31	
High school	8	12	
Degree	2	3	
**Crime 1 (** ***N*** **)**			3,417
Against life and integrity	10	13	
Against freedom	4	7	
Against property; public estate	46	52	
Against Public Health	8	20	
Gender violence	13	21	
**Crime 2(** ***N*** **)**			8,416
No crime	19	34	
Against life and integrity	15	10	
Against freedom	1	6	
Against property; public estate	34	40	
Against public health	10	16	
Gender violence	2	7	
**Alcohol and Drug Abuse History (** ***N*** **)**			10,487^**^
No consume	11	33	
Drug abuse	37	42	
Alcohol	4	12	
Alcohol and drug abuse	29	26	
**Drug Abuse (** ***N*** **)**			25,370^***^
Never	14	54	
Sometimes	33	39	
Frequently	22	9	
Always	6	7	
Very much	6	4	
**Alcohol abuse (** ***N*** **)**			8,216^*^
Never	22	37	
Sometimes	40	61	
Frequently	9	9	
Always	5	6	
Very much	5	0	
**Alcohol and drug quit treatment history (** ***N*** **)**			9,965^**^
Never	18	45	
Currently in prison	34	32	
Throughout life in prison	21	19	
Out of prison	8	17	

*** *p < 0.001*;

** *p < 0.01*;

* *p < 0.05*.

**Table 2:** Mean, Standard deviation.

**SCL-90-R**	**ASPD**	**OCPD**	***F***	**η**
	**Mean (SD)**	**Mean (SD)**		
Total SCL-90	4.34 (2.63)	38.12 (19.11)	0.596	0.003
Total positives symptoms	52.83 (23.85)	49.20 (23.01)	1.141	0.006
Index symptomatic distress	25.99 (18.87)	27.08 (19.60)	0.151	0.001
Somatizations	37.59 (23.98)	4.86 (25.06)	0.831	0.004
Obsessions and compulsions	44.69 (21.25)	42.88 (21.52)	0.339	0.002
Interpersonal sensitivity	42.65 (22.45)	42.96 (2.28)	0.010	0.000
Depression	41.57 (2.00)	43.54 (19.19)	0.478	0.002
Anxiety	4.03 (2.73)	35.42 (2.10)	2.418	0.012
Hostility	51.67 (2.26)	37.34 (14.22)	33.475^***^	0.148
Phobic anxiety	4.18 (15.49)	42.46 (16.82)	0.918	0.005
Paranoid Ideation	55.34 (17. 60)	54.33 (18. 18)	0.149	0.001
Psychoticism	47.78 (15. 44)	45.15 (16. 51)	1.257	0.007

*Significance level and statistical power of The Symptom Checklist (SCL-90-R)*.

*** *p < 0.001; ns = not significant*.

### Procedure

Potential participants were interviewed individually to check whether they meet the inclusion criteria, after which they were offered the opportunity to participate in the research. After agreeing to participate, they completed the IPDE (Loranger et al., 1994) and the Symptom Inventory Checklist (SCL-90-R; Derogatis and Savitz, 2002). Based on these results, participants with ASPD and OCPD were then selected. The participants then underwent an individual session in which they completed the measures described below. At the beginning of the session, the participants were reminded of their right to abandon the study at any moment and were asked to sign a written informed consent form if they agreed to participate. At the end of the session, participants were debriefed and thanked for their participation. All participants were informed about the aims of the study and provided written informed consent. Ethical approval for this study was obtained from the Research Ethics Committee of the Regional Government of Andalusia.

### Measures

#### Demographic, Crime, and Institutional Behavior Interview

The interview was designed specifically for this project with the aim of gathering socio-demographic data, information regarding the types of crimes committed and any punishment or prison sentences received according to the Spanish justice system (Royal Decree 1201/1981, 8 May, Articles 107 & 108).

#### International Personality Disorder Exam

(IPDE: Loranger et al., 1994; Spanish version developed by López-Ibor et al., 1996). This is a diagnostic instrument based on a semi-structured clinical interview, designed according to DSM-5 criteria [American Psychiatric Association (APA), 2013]. The items consist of open questions, multiple-choice questions, and yes/no questions. The items are classified according to the following six categories: work, self, interpersonal relations, affection, reality check, and impulse control. In addition, the IPDE includes a screening questionnaire that reduces the interview administration time by identifying the personality disorders that the person is unlikely to suffer from and then excluding further questions regarding these disorders. The administration of the IPDE takes between 60 and 90 min and must be carried out by trained and experienced professionals. The reliability and stability indices obtained for the IPDE vary between 0.70 and 0.96 (Loranger et al., 1994). The instrument is considered one of the most useful and valid tools for assessing personality disorders for research purposes (López-Ibor et al., 1996).

#### The Symptom Checklist-90-R (SCL-90-R)

This is a symptom scale developed by Derogatis (1994) that evaluates the degree of psychological distress a person has experienced in the past week. It consists of 90 items (or 52 in the reduced version) using Likert scales with five response options. The instrument is structured according to nine primary dimensions: somatizations (SOM), obsessions and compulsions (OBS), interpersonal sensitivity (IS), depression (DEP), anxiety (ANS), hostility (HOS), phobic anxiety (FOB), paranoid ideation (PAR), and psychopathy (PSIC). There are seven additional items targeting sleep disorders, eating disorders, death-related thoughts, and feelings of guilt. The following three global indices of distress are derived from these scales: the Index of Global Severity (IGS) indicating current levels of perceived distress, Total Positive Symptoms (TPS) indicating the total number of present symptoms, and the Index of Positive Symptomatic Distress (PSD) evaluating the response style toward symptoms. Reliability studies show that the nine dimensions reach values close to or greater than α = 0.70 and the concurrent and predictive validity of the inventory and its subscales have been confirmed, using as criteria other clinical evaluation instruments, screening scales, psychiatric diagnoses, structured evaluation protocols, or recidivism indicators (Derogatis and Savitz, 2002). We used the Spanish adaptation of the inventory (González-de Rivera et al., 2002).

#### Battery for Evaluation of Writing Processes (PROESC)

This is an individually applied test, created by Cuetos Vega et al. (2004), that aims to evaluate the main processes involved in writing and error detection. It consists of the following four subtests: (1) Dictation of Syllables; (2) Dictation of Words; (3) Dictation of Pseudowords; and (4) Dictation of phrases. It evaluates the following six aspects: Mastery of the phoneme-grapheme conversion rules; Knowledge of arbitrary spelling or lexical spelling; Command of spelling rules; Mastery of the rules of accentuation; Use of capital letters; and Use of punctuation marks. The manual of the instrument (Cuetos Vega et al., 2004) reports an internal consistency of 0.82 (coefficient alpha).

## Results

To address our study hypotheses, we proceeded to check whether the writing processes evaluated through PROESC differed between the groups. To do this, a Multivariate Analysis of Variance (MANCOVA) was carried out, for a between-group unifactorial design, using educational level as a covariate; group (ASPD and OCPD) as the independent variable, and the variables derived from the Battery for the Evaluation of the Writing Processes in the Dictation mode (Syllables, Words with Lexical Spelling, Words with Spelling Rules, Pseudowords, Pseudowords with Rule-based Spelling, Accent Phrases, Capital Phrases, Punctuation Mark Phrases) as dependent variables. This analysis revealed statistically significant differences between the groups [Wilks' Lambda = 0.237, *F*_(8, 184)_ = 73.962; *p* < 0.001].

Given that the MANCOVA showed a statistically significant main effect of group, we conducted univariate ANCOVAs for each of the levels of the dependent variable (Syllables, Words with Lexical Spelling, Words with Ruled-based Spelling, Pseudowords, Pseudowords with Spelling Rules, Accent Phrases, Capital Phrases, Punctuation Phrases). These ANCOVAs revealed statistically significant differences for Syllables [*F*_(2, 191)_ = 5.647; Mce = 62.136; *p* < 0.004], the scores being higher for the ASPD group than for the OCPD group; for Words with Lexical Spelling [*F*_(2, 191)_ = 18.406; Mce = 311.671; *p* < 0.001] with the ASPD group showing lower scores than the OCPD group; for Words with Rule-based Spelling [*F*_(2, 191)_ = 13.958; Mce = 247.862; *p* < 0.001] with the ASPD group showing higher scores than the OCPD group; for Pseudowords [*F*_(2, 191)_ = 8.271; Mce = 131.034; *p* < 0.001], with the ASPD group showing higher scores than the OCPD group; for Pseudowords with Rules-based Spelling [*F*_(2, 191)_ = 7.281; Mce = 49.552; *p* < 0.001] with the ASPD group showing higher scores than the OCPD group; for Accent Phrases [*F*_(2, 191)_ = 12.064; Mce = 248.984; *p* < 0.001] with the ASPD group showing higher scores than the OCPD group; for Capital Phrases [*F*_(2, 191)_ = 8.532; Mce = 90.185; *p* < 0.001] with the ASPD group showing higher scores than the OCPD group; for phrases with Punctuation Marks [*F*_(2, 191)_ = 23.589; Mce = 185.664; *p* < 0.001] with the ASPD group showing higher scores than the OCPD group (see Table 3).

**Table 3:** PROESC: Mean, standard deviation, significance level, and statistical power of writing (PROESC) of the groups.

**PROESC**	**ASPD**	**OCPD**	***F***	**η**
	**MEAN (SD)**	**MEAN (SD)**		
Syllables	20.01 (3.35)	19.40 (3.42)	5.647^**^	0.056
Words with lexical spelling	18.90 (4.02)	18.98 (4.79)	18.406^***^	0.162
Words withrule-based spelling	19.69 (3.72)	19.15 (4.97)	13.958^***^	0.128
Pseudowords	16.12 (3.75)	15.45 (4.38)	8.271^***^	0.080
Pseudowords with rule-based spelling	9.30 (2.42)	8.78 (2.86)	7.281^**^	0.071
Accent phrases	4.05 (4.70)	4.02 (4.89)	12.064^***^	0.112
Capital phrases	9.23 (2.88)	8.26 (3.65)	8.532^***^	0.082
Sentences punctuations marks	3.98 (3.11)	3.76 (3.13)	23.589^***^	0.198

*** *p < 0.001*;

** *p < 0.01*.

## Discussion

In the present study we evaluated writing disorders among a sample of impulsive and compulsive prisoners. In particular, we sought to confirm the original observation of an orthographic lexical disorder in OCPD prisoners, which could be similar to dyslexia. First, the OCPD group showed lower scores on Syllable Dictation in comparison with the ASPD group. For example, they have written, in Spanish, the syllable /wi/ instead of /güi/, or /zoo/ instead of /zo/ creating a real word from a syllable. Many OCPD prisoners added accents to the syllables and were doubtful with regard to the use of the /h/ at the beginning of the syllable. Moreover, most of the OCPD prisoners eliminated the last /-s/ or even added it in other cases, whilst they also changed /-l/ to /-r/, or /ch-/ to /x-/. Although these findings have never been described by other authors, Nicolson and Fawcett (2019), Nigro et al. (2015), and Zou (2017) claimed that many spelling rules are not highly familiar to people with dyslexia, and the same could be true for people with OCPD. Therefore, there are not studies that related OCPD to dyslexia, but the biological bases of compulsivity share multiple brain areas and neural circuits with language and communication (Ardila, 2016).

Second, the OCPD group showed higher scores on Words with Lexical Spelling in comparison with the ASPD group. This is a novel finding that could be explained by the characteristics of the OCPD profile. According to Nicolson and Fawcett (2019), this kind of tasks could represent a great challenge for dyslexia, on both adults and children. Even though many rules are not familiar to people with dyslexia, Nigro et al. (2015) suggested that, due to memory abilities, other spelling rules are sufficient to produce correct spellings and this could explain why the OCPD group performed better on this task.

Third, the OCPD group obtained lower scores on Words with Rule-based Spelling in comparison with the ASPD group, that is the OCPD group made spelling mistakes according to the basic rules of writing. For instance, they did not respect the Spanish writing rules regarding the use of /m-/ before /-p/ and /-b/, adding /-u-/ to the syllable /-gue/ for the proper spelling (/-ge/ instead of /-gue/). They also failed to correctly use the Spanish graphemes /y/, /j/ and /g/ (*injectar* for *inyectar*). These results are extremely novel, given that there are no recent studies that have examined spelling disorders in OCPD prisoners, although Afonso et al. (2015) and Suzuki and DeKeyser (2017) found that the word length effect affected dyslexics due to the cost of additional graphemic processing. In sum, it appears that people with OCPD and dyslexia make similar writing mistakes.

Fourth, the OCPD group obtained lower scores on Pseudowords and Pseudowords with Rule-based Spelling than the ASPD group. This finding could be explained by the fact that OCPD sufferers are strict and inflexible with orthographic rules or the OCPD group in our study have not acquired them (Cain et al., 2015). In addition, the results of this experiment suggest that the orthographic representation of new words or pseudowords is constructed through semantics and phonology, and we have already seen in the previous results (the OCPD group showed lower scores on Syllable Dictation and words with Rule-based Spelling compared with the ASPD group) that the OCPD group had great difficulties with rule-based structures. These pseudowords are easily transformable into words (for instance, “*olcho”* to “*ocho”* or “*zampeño”* to “*San Pedro*”). These results are congruent with those of Martínez-García et al. (2019) and Suzuki and DeKeyser (2017), indicating that semantic and phonological training could help with new words.

Fifth, regarding formal aspects, the OCPD group obtained lower scores on Accent Phrases, Capital Phrases and Phrases with Punctuation Marks than the ASPD group. This could be due to an alteration in certain cognitive elements of written composition such as the implementation of grammatical judgments and syntactic-semantic composition. These striking and novel findings are consistent with the results reported by Gutiérrez-Fresneda and Díez-Mediavilla (2017) who demonstrated that the main characteristics of dyslexia are related to the use of collocation and syntactic structure, along with formal aspects such as capital letters and punctuation marks.

In conclusion, prisoners with writing disorders are generally lacking the skills needed to write as a consequence of compulsive rather than impulsive behavior. OCPD is characterized by pervasive patterns of preoccupation with orderliness, along with perfectionism that is manifest in the preoccupation with details, rules, order, and organization. Moreover, OCPD sufferers show mental and interpersonal control at the expense of flexibility. This perfectionism could explain why people with OCPD perform behaviors or tasks in a recurring and repetitive way. These types of behaviors are also characteristics of dyslexia. Whilst no studies have been conducted to confirm this possibility, dyslexia and compulsivity share common biological bases (D'Mello and Gabrieli, 2018; Suhas and Rao, 2019). We have found that OCPD prisoners show many signs of dyslexia such as slow preparation and production of words, which is an accord with the study by Afonso et al. (2015) which confirmed that problems of slow and poor spelling in developmental dyslexia persist into adulthood.

It is important to acknowledge certain limitations of the present study. The first limitation could be due to the selection of men instead of a mixed gender sample, although we have evaluated crimes such as gender abuse, which is understood to mean male to female aggression, and the prison population contains five times more men than women. The second limitation is related to the measures used, which might involve the cognitive processes implied in language such as learning, attention, working memory, and executive functions. The third limitation is associated with the lack of dyslexia and control groups. In order to overcome these limitations, future research studies could include these new groups and could also attempt to evaluate the cognitive processes involved in language. However, the main strength of this study is that it is the first to analyse each part of the PROESC separately.

## Data Availability Statement

The raw data supporting the conclusions of this article will be made available by the authors, without undue reservation.

## Ethics Statement

The studies involving human participants were reviewed and approved by University of Granada. The patients/participants provided their written informed consent to participate in this study.

## Author Contributions

The main idea of this study and the exhaustive bibliographic search was developed by LM-L, FL-T, and FS. LM-L and FL-T took the samples from the participants. LM-L, FL-T, and FS wrote the manuscript and revised the text in its different versions. All authors participated in the statistical analysis.

## Conflict of Interest

The authors declare that the research was conducted in the absence of any commercial or financial relationships that could be construed as a potential conflict of interest.

## Publisher's Note

All claims expressed in this article are solely those of the authors and do not necessarily represent those of their affiliated organizations, or those of the publisher, the editors and the reviewers. Any product that may be evaluated in this article, or claim that may be made by its manufacturer, is not guaranteed or endorsed by the publisher.
